# Extracellular vesicle-associated microRNA signatures related to lymphovascular invasion in early-stage lung adenocarcinoma

**DOI:** 10.1038/s41598-023-32041-5

**Published:** 2023-03-24

**Authors:** Yoshihisa Shimada, Yusuke Yoshioka, Yujin Kudo, Takahiro Mimae, Yoshihiro Miyata, Hiroyuki Adachi, Hiroyuki Ito, Morihito Okada, Tatsuo Ohira, Jun Matsubayashi, Takahiro Ochiya, Norihiko Ikeda

**Affiliations:** 1 Department of Thoracic Surgery, Tokyo Medical University, 6-7-1 Nishishinjuku, Shinjuku-ku, Tokyo, 160-0023 Japan; 2 Department of molecular and cellular medicine, Tokyo Medical University, Tokyo, Japan; 3 Department of Anatomic Pathology, Tokyo Medical University, Tokyo, Japan; 4 Department of Surgical Oncology, Hiroshima University, Hiroshima, Japan; 5 Department of Thoracic Surgery, Kanagawa Cancer Center, Yokohama, Kanagawa Japan

**Keywords:** Lung cancer, Tumour biomarkers

## Abstract

Lymphovascular invasion (LVI) is a fundamental step toward the spread of cancer. Extracellular vesicles (EVs) promote cellular communication by shuttling cargo, such as microRNAs (miRNAs). However, whether EV-associated miRNAs serve as biomarkers for LVI remains unclear. This study aimed to identify EV-associated miRNAs related to LVI and validate the miRNA levels from patients with early-stage lung adenocarcinoma (LADC). Blood samples were collected from patients undergoing pulmonary resection for stage I LADC before surgery. The patients were classified into three groups according to the presence of LVI and postoperative recurrence. Serum-derived EVs in the derivation cohort were used for small RNA sequencing, while the selected LVI miRNA candidates were validated via real-time quantitative polymerase chain reaction using 44 patient and 16 healthy donor samples as the validation cohorts. Five miRNAs (miR-99b-3p, miR-26a-5p, miR-93-5p, miR-30d-5p, and miR-365b-3p) were assessed, and miR-30d-5p (*p* = 0.036) levels were significantly downregulated in the LVI-positive group. miR-30d-5p levels in healthy donors were lower than those in LADC patients. Patients with high miR-30d-5p levels had favorable survival compared to those with low miR-30d-5p levels. miR-30d-5p level in EVs may serve as a promising biomarker for detecting LVI in patients with early-stage LADC.

## Introduction

Pathological lymphovascular invasion (LVI), which is the presence of vascular invasion and/or lymphatic permeation, is a strong prognostic factor in many types of cancer, including lung cancer^1–7^. The presence of LVI, which reflects tumor aggressiveness, is an essential step toward locoregional and systemic tumor spread. Invasion and metastasis are the primary causes of death in patients with lung cancer. Therefore, understanding the regulatory mechanisms of LVI may help in overcoming lung cancer and developing new therapeutic targets.

MicroRNAs (miRNAs) are non-coding RNAs that regulate gene expression at the post-transcriptional level, and have emerged as promising biomarkers for cancer diagnosis and therapy^8,9^. miRNAs are transported via body fluids within extracellular vesicles (EVs), including exosomes^10–12^. EVs are bioactive vesicles that promote cell–cell communications by shuttling cargo, such as mRNAs, proteins, miRNAs, and lipids^13–15^. EVs have been widely studied, and cancer-derived EVs have been found to be involved in metastatic cascades, such as tumorigenesis, migration, priming of metastatic niches, and LVI^16–23^. Several studies have demonstrated the correlation between LVI and aberrant expression of specific miRNAs^24,25^. However, only a few studies have performed EV-associated miRNA profiling with small RNA sequencing. It remains unclear whether EVs containing unique miRNAs can serve as non-invasive tools for the early diagnosis and in-depth understanding of the mechanism of LVI in patients with early-stage lung cancer, especially lung adenocarcinoma, which is the most common type of non-small cell lung cancer (NSCLC).

This study aimed to identify specific EV-associated miRNAs related to LVI via small RNA sequencing and quantify their miRNA levels in serum EVs from patients with early-stage lung adenocarcinoma for validation analysis.

## Materials and methods

### Prognostic analysis according to LVI status using a large database

We used a clinical database from three institutions (Tokyo Medical University Hospital, Hiroshima University Hospital, and Kanagawa Cancer Center), including 4676 patients who underwent surgical resection for lung cancer between January, 2010 and December, 2020. Among them, 2044 patients with pathological stage I NSCLC undergoing radical anatomical resection (segmentectomy or lobectomy) between January, 2010 and December, 2016 were selected to assess postoperative survival outcomes according to LVI status. The TNM stage was determined according to the seventh edition of the TNM classification of malignant tumors.

### Research subjects

**Figure 1:**
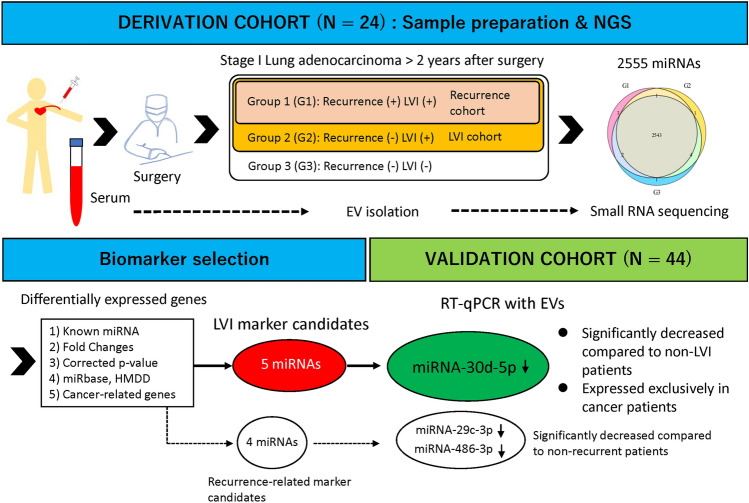
Schematic overview of the strategy used to identify extracellular vesicle-associated microRNA (miRNA) signature associated with vascular invasion and postoperative recurrence. *NGS* nest generation sequencing, *LVI* vascular invasion, *EV* extracellular vesicle, *miRNAs* microRNAs, *RT-qPCR* reverse transcription-quantitative polymerase chain reaction.

Blood samples were collected from 68 patients before surgery in the operating room. All patients underwent complete surgical resection for pathological stage I lung adenocarcinoma at the Tokyo Medical University Hospital between January, 2015 and December, 2019. The tumor specimens were fixed in neutral buffered formalin, processed routinely and embedded in paraffin and serially sectioned at 4-μm thickness and stained with hematoxylin/eosin and Elastica van Gieson to evaluate the extent of blood vessel invasion and pleural invasion. The sections were also stained with D2-40 to evaluate the extent of lymphatic permeation. The patients were classified into three groups based on the presence of LVI and postoperative recurrence. The derivation set included serum-derived EVs from 24 blood samples used for small RNA sequencing, while 44 blood samples were used for the validation study. Selected LVI and recurrence-related miRNA candidates from small RNA sequencing were subsequently validated using reverse transcription-quantitative polymerase chain reaction (RT-qPCR) in the validation cohort. A schematic overview of the study is shown in Fig. 1. Serum samples from 16 healthy donors were also collected as controls for RT-qPCR. Each blood sample was centrifuged at 3000 rpm for 5 min to separate the serum, which was stored at – 80 °C until RNA extraction. This study was approved by the institutional review board of Tokyo Medical University (study approval no. T2021-0140). Written informed consent for the use and analysis of clinical data was obtained preoperatively from each patient and healthy donor. All experiment protocols were approved by the Institutional Review Board of Tokyo Medical University, and all methods were carried out in accordance with relevant guidelines and regulations.

### EV isolation

A sequential centrifugation procedure was used to recover EVs. Venous blood from each patient was separated into serum and cellular fractions. Cells were pelleted via centrifugation at 300×*g* for 5 min, followed by centrifugation at 1200×*g* for 20 min. To eliminate other cellular debris, the supernatant was centrifuged at 10,000×*g* for 30 min. For EV preparation, the sample was ultracentrifuged at 210,000×*g* for 35 min at 4 °C. Pellets were washed with phosphate-buffered saline.

### Small RNA library preparation and sequencing

Total EV-associated miRNA of 24 samples in the derivation set was isolated using the miRNeasy Mini Kit (QIAGEN, The Netherlands) and sent to DNA Chip Research Inc., where small RNA library preparation and sequencing were performed. Small RNA libraries were prepared using the QIAseq miRNA library kit and QIAseq miRNA NGS 96 Index IL for Illumina, and yields were evaluated using the Agilent 2100 BioAnalyzer. Small RNA sequencing was performed using the Illumina NexSeq 500 system.

### Identification of LVI and recurrence-related miRNAs

EV-associated miRNA selection criteria from small RNA sequencing data included known miRNAs and differentially expressed genes (DEGs) that were obtained based on the criteria of |log2 fold changes|> 1.5, and corrected p-value by Benjamini–Hochberg false discovery rate controlling procedure of less than 1. The miRNA databases, miRBase (https://www.mirbase.org) and HMDD (https://www.cuilab.cn/hmdd) were used to identify the LVI and recurrence-related miRNAs associated with cancer.

### RT-qPCR

We performed RT-qPCR analysis of selected EV-associated miRNAs related to LVI in the validation cohort to validate the small RNA sequencing data. EV-associated miRNAs were isolated using the miRNeasy Mini Kit and cDNAs were generated using the TaqMan MicroRNA Reverse Transcription Kit (Thermo Fisher Scientific, Waltham, Massachusetts). Gene-specific TaqMan MicroRNA Probes (Thermo Fisher Scientific) were used for quantitative analyses of the miRNA transcript levels of miR-99b-3p, miR-26a-5p, miR-93-5p, miR-30d-5p, miR-365b-3p, miR-29c-3p, miR-486-3p, miR-486-5p, and miR-548ae-5p. To normalize miRNA expression, let7f was selected as an internal control in our experiment because the gene as an endogenous control miRNA has been reported to be stable during senescence and aging^26^.

### Statistical analyses

Statistical analyses were performed using the Statistical Analysis Software Package R (R Project for Statistical Computing; http://www.r-project.org) and the SPSS statistical software package (version 28.0; DDR3 RDIMM; SPSS Inc., Chicago, IL, USA). Overall survival (OS) was measured from the day of surgery to the day of death from any cause or the day on which the patient was last known to be alive. Recurrence-free survival (RFS) was measured as the interval between the date of surgery and the date of recurrence, the date of death from any cause, or the date on which the patient was last known to be alive. OS and RFS curves were plotted using the Kaplan–Meier method, and differences in variables were determined using the log-rank test. For survival analysis in the validation cohort, we excluded those with a follow-up period of less than 1 year and those who died of adverse events or non-cancer-related death within 1 year after surgery. Univariate and multivariate logistic regression analyses were performed to identify the factors associated with OS and RFS using a Cox proportional hazards model. A backward stepwise selection method was used to build the logistic regression models, and variables with a threshold of *p* < 0.15 were adopted for the stepwise model selection procedure to prevent overlooking relevant factors^27^. Student’s *t* test for continuous data was used to compare the two groups. All tests were two-sided, and *p*-values < 0.05 were considered to be statistically significant.

## Results

### Significance of LVI for survival in early-stage NSCLC

**Figure 2:**
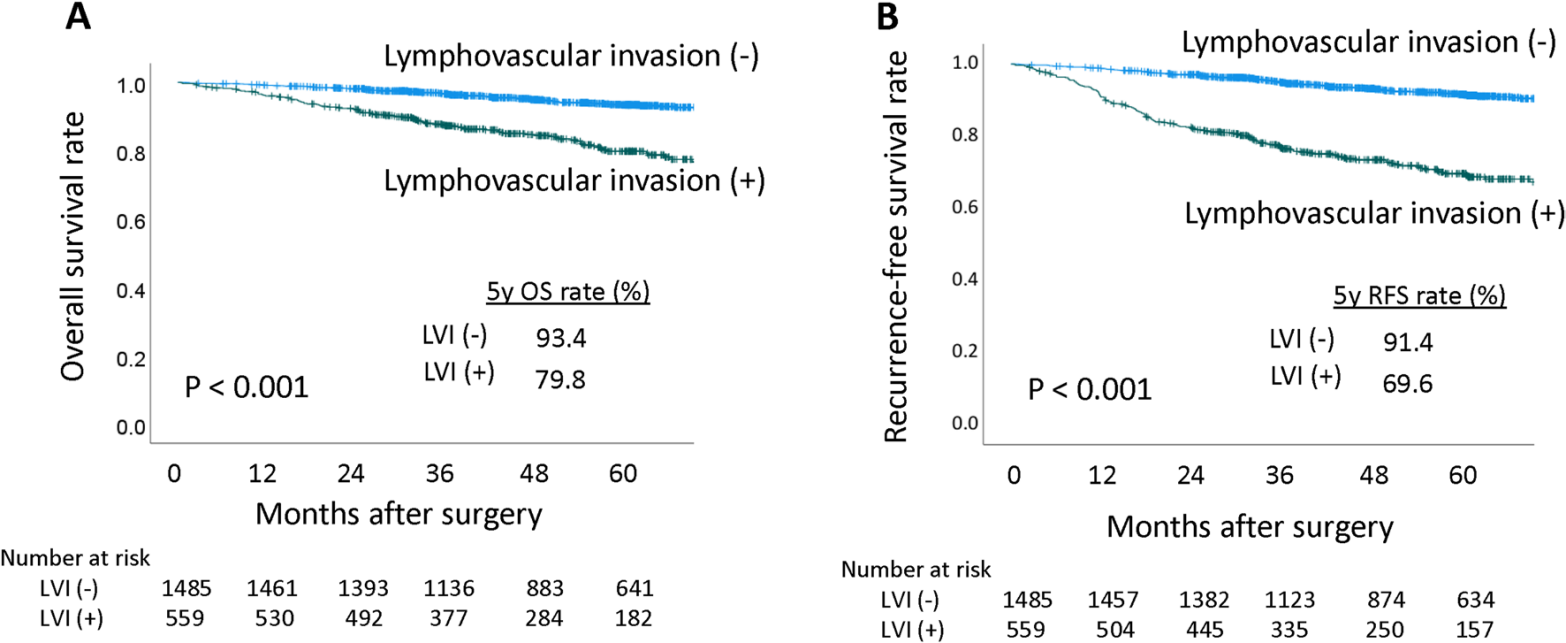
(**A**) Overall survival curves of patients with vascular invasion ( +) and vascular invasion ( −). (**B**) Recurrence-free survival curves of patients with vascular invasion ( +) and vascular invasion (–). *OS* overall survival, *LVI* vascular invasion, RFS recurrence-free survival.

Among 2044 patients with pathological stage I NSCLC in our database, 559 (27.3%) were found to be LVI-positive. Characteristics of the patients from our database was shown in Supplementary Table 1. OS and RFS were significantly worse in patients with LVI than in those without LVI (5-year OS rates of 93.4 vs. 79.8%, *p* < 0.001, Fig. 2A; 5-year RFS rates of 91.4 vs. 69.6%, *p* < 0.001, Fig. 2B). Multivariate analysis showed that maximum standardized uptake value (SUVmax) of tumor (hazard ratio [HR], 1.043; 95% confidence interval CI 1.008–1.079; *p* = 0.017), pathological tumor size, (HR, 1.357; 95% CI 1.060–1.733; *p* = 0.015), pathological stage (HR, 2.427; 95% CI 1.428–4.124; *p* = 0.001), and LVI (HR, 4.134; 95% CI 2.431–7.031; *p* < 0.001) were independent risk factors for OS (Supplementary Table 2), while age (HR, 1.048; 95% CI 1.032–1063; *p* < 0.001), smoking history (HR, 1.657; 95% CI 1.140–2.408; *p* = 0.008), SUVmax of tumor (HR, 1.024; 95% CI, 1.004–1.044; *p* = 0.020), surgical procedure (HR, 1.563; 95% CI 1.195–2.045; *p* = 0.001), pathological stage (HR, 1.771; 95% CI 1.350–2.323; *p* < 0.001), and LVI (HR, 2.664; 95% CI 2.000–3.548; *p* < 0.001) were the significant risk factors for RFS (Supplementary Table 3).

### Characteristics of the research population

In the research population, there were 24 patients with LVI and postoperative recurrence in group 1, 22 patients with only LVI in group 2, and 22 patients without LVI and recurrence in group 3. The general patient characteristics are shown in Table 1. The numbers of patients in the derivation and validation sets were 24 and 44, respectively. A significant difference was observed between patients with LVI (G1 + 2) and those without LVI (G3) in terms of smoking history (*p* = 0.006), pathological stage (*p* = 0.002), presence of pleural invasion (*p* < 0.001), and tumor differentiation (*p* < 0.001). In contrast, there was no significant difference between G1 and G2, except for the pathological tumor size (*p* = 0.037).

**Table 1:** Characteristics of patient groups (n = 68).

Factors	Group 1, LV + /R + n = 24 (%)	Group 2, LV + /R- n = 22 (%)	Group 3, LV-/R− n = 22 (%)	p-value, G 1 + 2 vs. 3	p-value, G 1 vs. 2
Age, mean ± SD	67 ± 10	68 ± 9	69 ± 10	0.637	0.454
Sex					
Male	12 (50)	14 (64)	7 (32)	0.068	0.351
Female	12 (50)	8 (36)	15 (68)		
Smoking history, yes	16 (67)	17 (77)	8 (36)	0.006	0.425
Pathological stage					
IA	2 (8)	6 (27)	12 (55)	0.002	0.090
IB	22 (92)	16 (73)	10 (46)		
Vascular invasion, yes	24 (100)	22 (100)	0	–	–
Pleural invasion. yes	15 (63)	11 (52)	1 (5)	< 0.001	0.493
Tumor differentiation					
Well	2 (8)	3 (14)	15 (68)	< 0.001	0.473
Moderate	20 (83)	15 (68)	7 (32)		
Poor	2 (8)	4 (18)	0		
Pathological tumor size, cm, mean ± SD	3.6 ± 2.1	2.7 ± 0.9	2.8 ± 1.8	0.402	0.037
Recurrence, yes	24 (100)	0	0	–	–
Dataset					
Derivation cohort	8 (33)	8 (36)	8 (36)	–	–
Validation cohort	16 (67)	14 (64)	14 (64)		

*LV* lymphovascular invasion, *R* recurrence within 2 years after surgery, *SD* standard deviation.

### Identification of LVI and recurrence-related EV-associated miRNAs in the two cohorts

**Figure 3:**
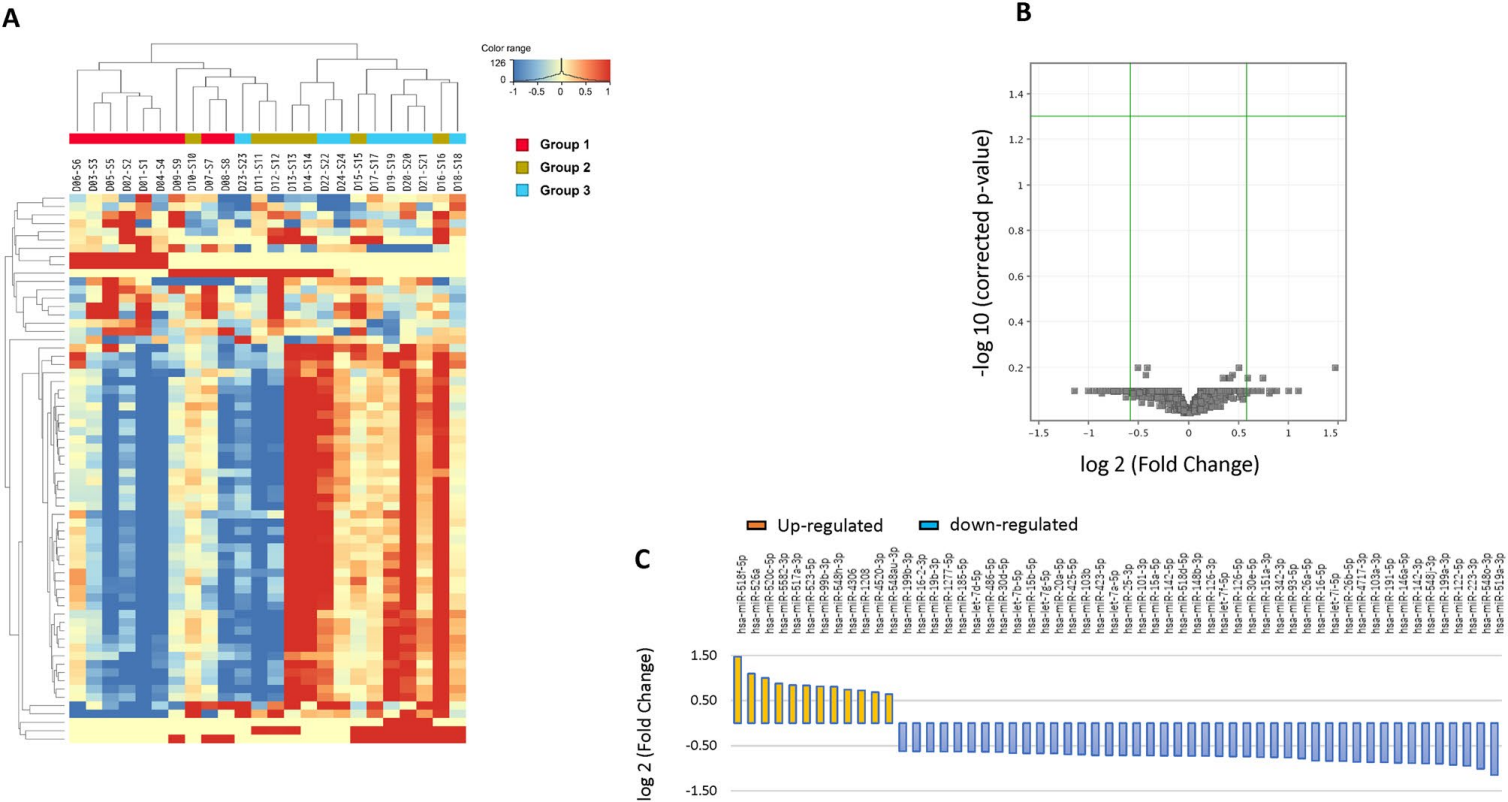
(**A**) Hierarchical clustering heatmap of 24 samples from patients with stage I lung adenocarcinoma. (**B**) Volcano plot of differentially expressed miRNAs between the G1 + 2 (LVI +) and G3 (LVI–) cohorts. (**C**) Charts indicate the fold-change expression of 66 miRNAs obtained as LVI marker candidates.

Expression levels of 2555 miRNAs were determined via small RNA sequencing. The miRNA levels of DEGs between patients with LVI (G1 + 2) and those without LVI (G3) are shown as a heatmap in Fig. 3A. As shown in the volcano plot, miRNAs were differentially expressed between the two cohorts (Fig. 3B). The criteria of |log2 fold changes|> 1.5, corrected p-value < 1, and being known miRNAs were used to identify the LVI-associated miRNAs, and 56 miRNAs were selected (Fig. 3C). Because let7f was selected as the internal control for RT-qPCR, let7 family miRNAs were excluded from the 56 miRNAs. We then used the miRNA databases, miRbase and HMDD, searchable databases for published miRNA sequences and annotation data, to narrow down the cancer-related candidate miRNAs, and 17 miRNAs were finally selected. Likewise, the miRNA levels of DEGs between patients with LVI but no recurrence (G2) and those with both factors (G1) are shown in Supplementary Fig. 1A. The volcano plot shows the differentially expressed miRNAs between the two cohorts (Supplementary Fig. 1B). We extracted 79 miRNAs using the same criteria as those for the LVI marker candidates (Supplementary Fig. 1C), and 14 miRNAs were ultimately selected.

### The exploration of miRNA associated with LVI and recurrence

**Figure 4:**
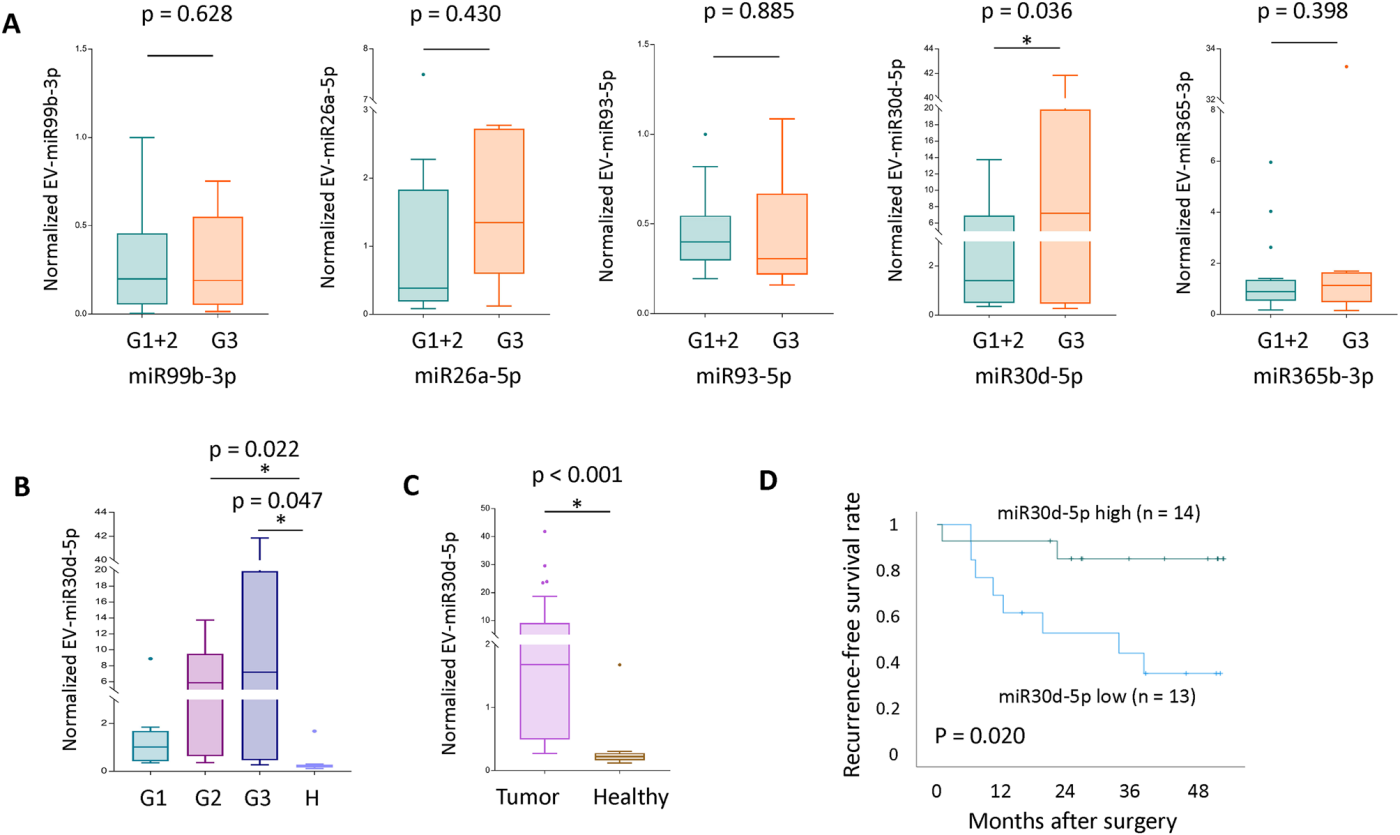
(**A**) Quantification of five miRNAs obtained from the serum extracellular vesicles of 44 patients for a validation study. G1 + 2 (LVI +) cohort has significantly lower miR-30d-5p levels (*p* = 0.036) than the G3 (LVI–) cohort. (**B**) miR-30d-5p levels in the healthy control group were significantly lower than those in the G2 (*p* = 0.022) and G3 (*p* = 0.047) cohorts. (**C**) miR-30d-5p levels in the healthy control group were significantly lower than those in the patients with lung adenocarcinoma (*p* < 0.001). (**D**) Recurrence-free survival curves of 27 patients with stage I lung adenocarcinoma according to their miR-30d-5p levels. *EV* extracellular vesicle.

Of the 17 miRNAs related to LVI, we excluded 12 genes (miR-519a-3p, miR-518d-5p, miR-520c-5p, miR-526a, miR548o-3p, miR-518f-5p, miR-191-5p, miR-486-5p, miR-223-3p, miR-16-5p, miR-146a-5p, miR-142-3p) due to undetermined gene expression levels via RT-qPCR. Thus, we selected the remaining five miRNAs (miR-99b-3p, miR-26a-5p, miR-93-5p, miR-30d-5p, and miR-365b-3p) for RT-qPCR confirmation in the validation cohort. Patients with LVI had significantly lower miR-30d-5p levels (*p* = 0.036) than those without LVI (Fig. 4A). In the other validation study with healthy control groups, the miR-30d-5p levels in the healthy control group were significantly lower than those in patients with G2 (*p* = 0.022; Fig. 4B) and G3 (*p* = 0.047; Fig. 4B), as well as those with lung adenocarcinoma (*p* < 0.001; Fig. 4C). To test the prognostic significance of serum EV miR-30d-5p level dichotomized at its median value, Kaplan–Meier curves of 27 patients with stage I lung adenocarcinoma showed that those with high miR-30d-5p levels had significantly favorable RFS compared to those with low miR-30d-5p levels (Fig. 4D).

Of the 14 miRNAs related to recurrence, we excluded 10 miRNAs (miR-519a-3p, miR-520c-3p, miR-523-5p, miR-548ah-3p, miR-642b-3p, miR-548 h-3p, miR-520c-5p, miR-92a-3p, miR-519e-3p, miR-517c-3p) due to undetermined gene expression levels via RT-qPCR. Therefore, we selected four miRNAs (miR-29c-3p, miR-486-3p, miR-486-5p, and miR-548ae-5p) for RT-qPCR confirmation in the validation cohort. Patients who experienced postoperative recurrence had significantly lower miR-29c-3p (*p* = 0.011) and miR-486-3p (*p* = 0.040) levels than those without recurrence (Supplementary Fig. 2). However, the expression level of these two miRNA in healthy donors was higher than that in patients with lung adenocarcinoma (miR-29c-3p, *p* = 0.013; and miR-486-3p, *p* = 0.121, data not shown).

### Enrichment and pathway analyses of EV-associated miRNAs related to LVI and recurrence

**Figure 5:**
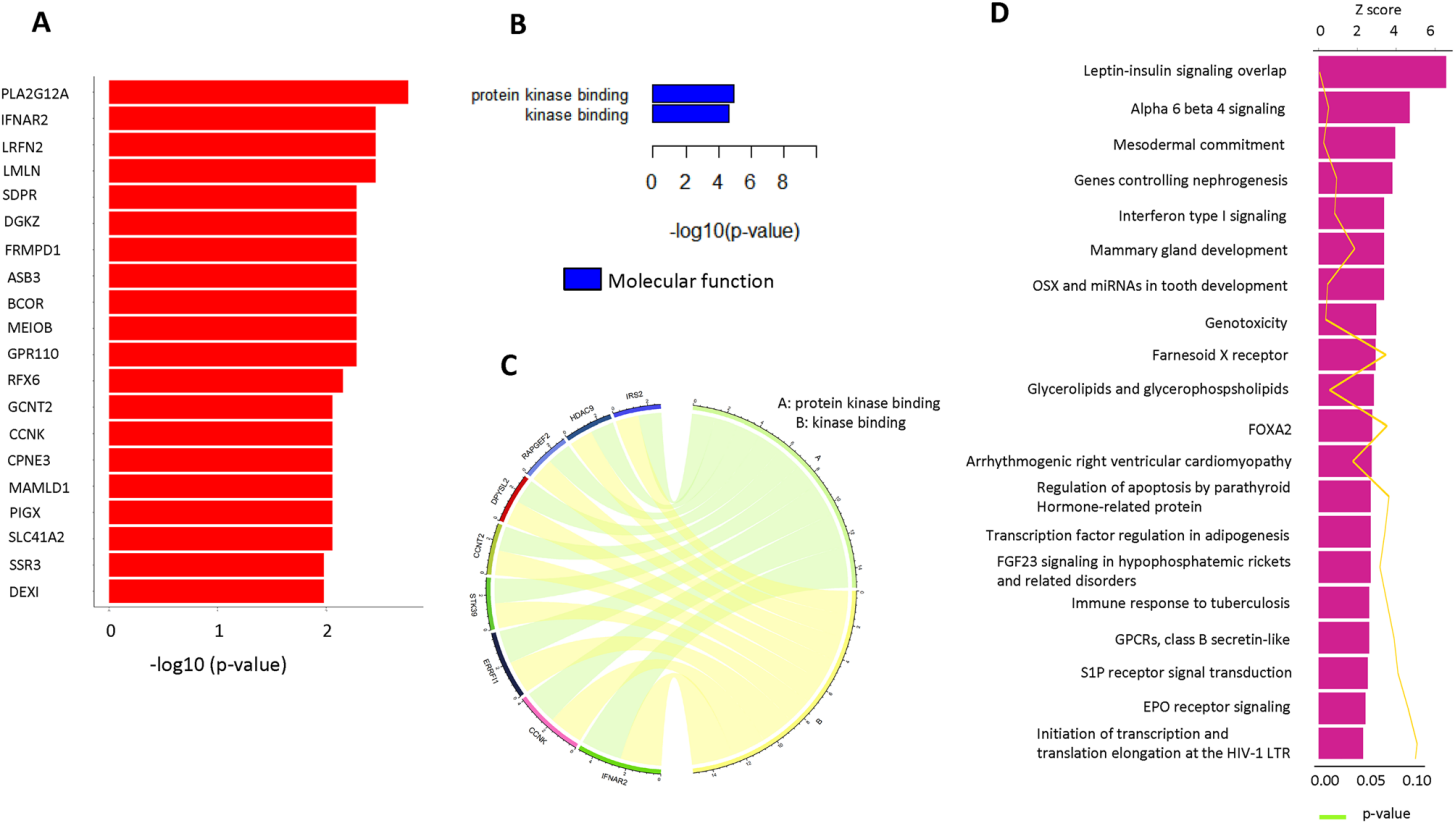
(**A**) List of the top 20 genes with strong correlations with miR-30d-5p target genes based on the *p*-value. (**B**) Two enriched Gene Ontology (GO) terms in molecular functions were correlated with the miR-30d-5p target genes. (**C**) Chord diagram illustrates the interaction between GO terms and the relevant gene symbols. (**D**) Top 20 pathways for target genes associated with miR-30d-5p based on the Z-score.

To fully determine the roles of miR-30d-5p in LVI and miR-29c-3p and miR-486-3p in recurrence, we collected statistically significant target genes from three databases, PITA, TargetScan, and microRNA.org., and performed Gene Ontology (GO) term and pathway annotation using WikiPathways and PathVisionRPC v1.2. Among the 69 significant targeted genes for miR-30d-5p, a list of the top 20 genes with strong correlations based on *p*-values is shown in Fig. 5A. GO enrichment analysis showed that two enriched GO terms, protein kinase binding and kinase binding in molecular functions, were correlated with the miR-30d-5p target genes (Fig. 5B). A chord diagram illustrates the interaction between GO terms and relevant gene symbols (Fig. 5C). The pathway analysis results showed that the target genes for miR-30d-5p were significantly related to leptin–insulin signaling overlap and alpha 6 beta 4 signaling (Fig. 5D). The *Z*-score of the most enriched pathways was greater than two, indicating that most of the pathways were enhanced.

There were 67 and 369 significant target genes for miR-29c-3p and miR-486-3p, respectively, and a list of the top 20 target genes is shown in Supplementary Fig. 3A. Six enriched GO terms correlated with miR-29c-3p target genes and the top 10 GO terms associated with miR-486-3p targets in three main categories (biological process, molecular function, and cellular component) are shown in Supplementary Fig. 3B. Chord diagrams illustrate the interaction between GO terms and relevant gene symbols in miR-29c-3p and miR-486-3p target genes (Supplementary Fig. 3C). Top 20 pathways for the target genes associated with the two recurrence-related miRNAs, based on the Z-score, are shown in Supplementary Fig. 3D.

## Discussion

The present study found that LVI is a prominent prognostic factor in a large number of patients with stage I NSCLC who underwent complete surgical resection. EV-associated miRNA profiling via small RNA sequencing identified miR-30d-5p to be associated with LVI using serum EVs derived from patients with stage I lung adenocarcinoma. Validation analysis using RT-qPCR demonstrated significant downregulation of EV-associated miR-30-5p levels in the LVI cohort, and gene expression was confirmed exclusively in tumors. Kaplan–Meier curves for RFS showed that miR-30d-5p was a prognostic marker in patients with early-stage adenocarcinoma. Finally, enrichment and pathway analyses of miR-30d-5p identified the correlated targeted genes, relevant enriched GO terms, and potential pathways involved in the occurrence of LVI in lung cancer.

LVI has prognostic significance in patients with resected lung cancer, especially adenocarcinoma, and reflects the tumor aggressiveness^1–7^. Local tumor invasion and entry of tumor cells into the vasculature occur at the early-stage of the metastatic cascade. Therefore, this phenomenon is often observed in patients with early-stage lung cancer without lymph node metastasis, and a better understanding of the regulatory mechanism of LVI may lead to the development of novel prognostic markers and effective therapeutic strategies to prevent the spread of metastasis. Complex cancer tissue components, such as immune cells, cancer-associated fibroblasts, and endothelial cells, contribute to the formation of a tumor microenvironment, which is involved in tumor cell intravasation and angiogenesis^16,18,21,28–30^. However, the mechanism by which cancer cells modulate the surrounding cells to facilitate LVI and the specific genetic determinants involved in cancer cell intravasation remain poorly understood.

Cancer-derived EVs contribute to the modulation of a tumor microenvironment favorable to cancer cells via cell–cell communication^21,22,31^. They play various roles in promoting tumor progression, invasion, migration, angiogenesis, and establishment of the pre-metastatic niche^15,18,19^. Cancer-derived EVs modulate endothelial cells, which play roles in intravasation, angiogenesis, loss of the endothelial vascular barrier, and extravasation, for vascular-related functions^24,25,29,32^. EVs contain a large number of miRNAs, and EV-associated miR-140-3p, miR-30d-5p, miR-29b-3p, miR-130-3p, miR-330-5p, and miR-296-3p have been reported to be associated with the migration ability of hepatocellular cells via comparative analysis of EV miRNA profiles^33^. Mao et al. reported that tumor-derived miR-494 promoted angiogenesis, and miR-21 transferred via EVs was taken up by the endothelial cells in their lung cancer study^25^. However, there is a lack of reports that patients’ serum derived EVs containing unique miRNAs that can serve as non-invasive biomarkers for LVI in early-stage lung adenocarcinoma.

Here, the validation dataset demonstrated that miR-30d-5p levels in serum EVs from LVI-negative patients were significantly higher than those from LVI-positive patients and healthy donors. miR-30d-5p has been reported to act as a tumor suppressor in various types of cancer, but only a limited number of studies have reported its role as an oncogene^27,34,35^. Gao et al. reported that miR-30d-5p levels were decreased in NSCLC tissues, and patients with lower miR-30d-5p levels tended to show an advanced clinical progression^36^. Chen et al. demonstrated that miR-30-5p levels were downregulated in NSCLC tissues, and that the gene and its direct target, cyclin E2 axis, may contribute to NSCLC proliferation and motility^35^. Zheng et al. showed that miR-30d-5p was a significant diagnostic biomarker for cervical cancer and its precursors^34^. Hu et al. demonstrated that the upregulation of four miRNAs, including miR-30d and miR-486, was significantly associated with poor OS using a large number of NSCLC samples^37^. The opposite results between their study and ours may be explained by differences in sample size, patients backgrounds, the lack of standardization for normalization, miRNA processing, the inability to discriminate among closely related miRNAs, and the source of miRNAs. Although the source of circulating miRNA is reported to be diverse, it can be inferred that exosomal miRNAs can act as a better source for biomarker studies because of its advantages in terms of quantity, quality, and stability by a systemic review^38^. In the current study, the level of exosomal miR-30d-5p in the healthy control group was significantly lower than that in samples from lung adenocarcinoma, suggesting that the biomarker can also aid in the diagnosis of early-stage lung adenocarcinoma on the purpose of a non-invasive early detection screening.

EV-associated miR-29c-3p and miR-486-3p levels from recurrent patients were significantly lower than those from non-recurrent patients. There are a small number of published studies related to the association between miR-29c-3p expression and cancer biology. The levels of miR-29c-3p were reported to be lower in colorectal cancer tissues than in non-cancerous tissues^39^. Ji et al. found that the miR-486-3p gene expression levels were downregulated in tumor tissues than in normal tissues in patients with hepatocellular carcinoma, and that it mediated sorafenib resistance by targeting the fibroblast growth factor receptor 4 and epidermal growth factor receptor^40^. Overall, these results suggest that patients with LVI-positive or recurrent lung adenocarcinoma may show significantly lower expression levels of these tumor suppressive genes than LVI-negative or non-recurrent patients and healthy donors.

Disrupting the intratumor vasculature and anti-angiogenic targeted therapy are key treatments for various types of tumors, including lung adenocarcinoma^41^. These therapies help starve cancer cells by halting angiogenesis and destroying pre-existing tumor-related vascular networks. Although several anti-vascular endothelial growth factor therapies have been well established, their effects are transient. Leptin-insulin signaling overlap and alpha 6 beta 4 signaling are the most significantly activated canonical pathways relating to miR-30d-5p in the current study. Leptin and insulin upregulate miR-4443 in colorectal cancer and decrease the invasiveness of colon cancer cells^42^. Alpha 6 beta 4 integrin signaling is reported to stimulate the invasion and migration of endothelial cells and promote angiogenesis^43^. Although there is no study to investigate miR-30d-5p and these cancer-related signaling, more in-depth gene expression profiling, and functional analysis may allow for understanding prognostic significance in malignancies including lung adenocarcinoma. Further research is required to investigate the roles of EV-associated miR-30d-5p in intravasation and angiogenesis in lung cancer, and to assess whether this liquid biopsy approach can be applied in daily clinical practice to predict postoperative survival and treatment efficacy.

Our study has several limitations. The small number of participants enrolled in this study could be a limitation; however, the inclusion of the validation cohort strengthened our results. The type of patients recruited may be a limitation; therefore, further research using larger and independent cohorts is warranted to validate the EV-associated miRNA signatures reported here. Finally, the methods of isolation and characterization of circulating EVs remain ambiguous. However, we have reported several other studies on EVs obtained using the same isolation methodology as serum-derived biomarkers.

In conclusion, our study found that EV-associated miRNA profiles associated with LVI target some critical pathways, suggesting the potential of an EV-associated miRNA, miR-30d-5p, as a non-invasive biomarker and therapeutic target to prevent cancer cell intravasation at the pivotal early step of the metastatic cascade.

## Supplementary Information

Supplementary Figures.

Supplementary Tables.

## Data Availability

The analyzed data in this article will be shared on reasonable request to the corresponding author.

## Abbreviations

LVI: Lymphovascular invasion
miRNA: MicroRNA
NSCLC: Non-small cell lung caner
RT-qPCR: Reverse transcription-quantitative polymerase chain reaction
DEG: Differentially expressed gene
EV: Extracellular vesicle
OS: Overall survival
RFS: Recurrence-free survival
HR: Hazard ratio
CI: Confidence interval
