# Supplementary material for: Extracellular vesicle-associated microRNA signatures related to lymphovascular invasion in early-stage lung adenocarcinoma
Source: Sci Rep. 2023 Mar 24;13:4823. doi: 10.1038/s41598-023-32041-5 (PMC10038982; doi:10.1038/s41598-023-32041-5)
Supplement: Supplementary file 1 — Supplementary Figures. [file 41598_2023_32041_MOESM1_ESM.pptx]

## Slide 1
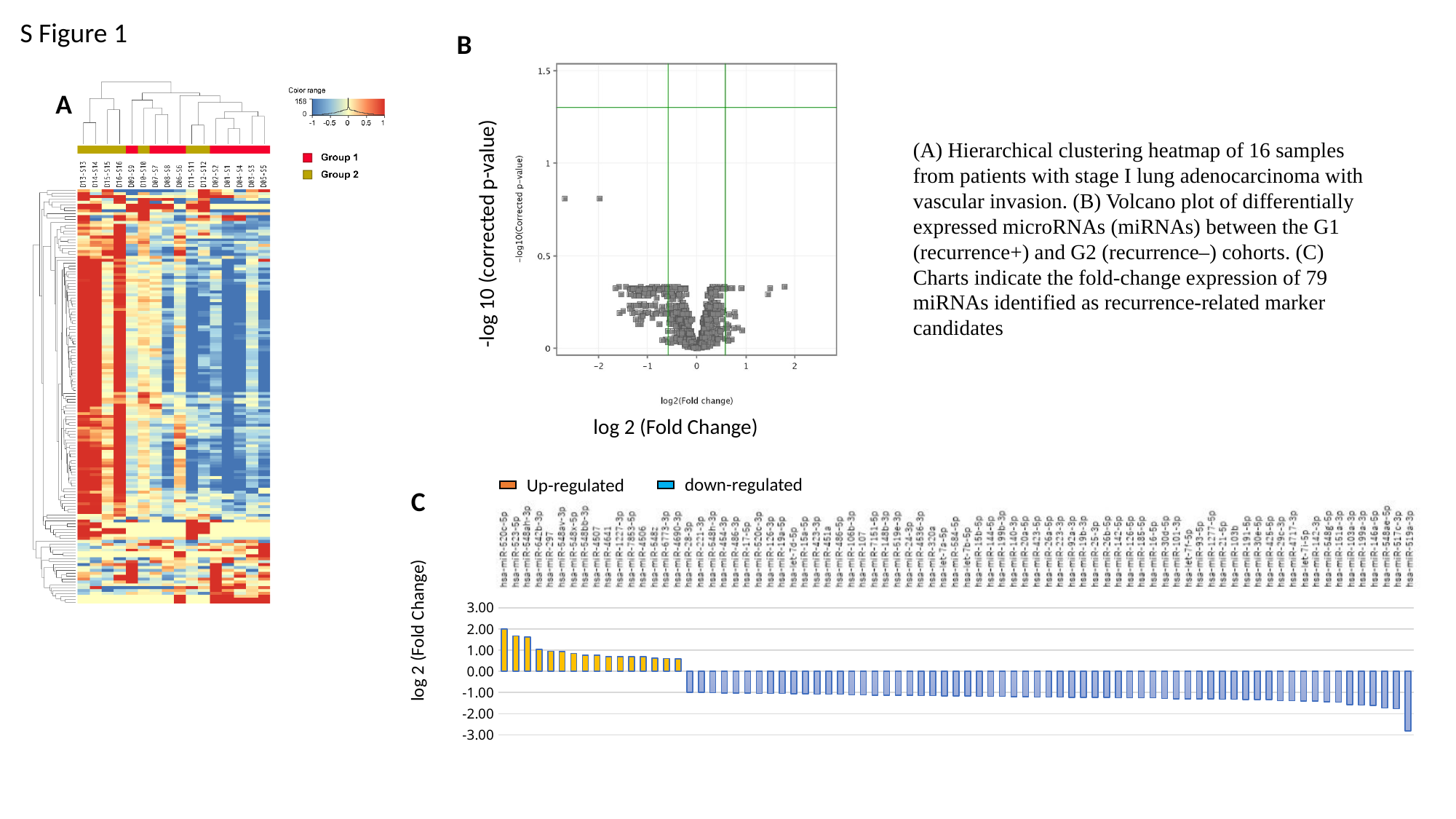

S Figure 1
B
-log 10 (corrected p-value)
log 2 (Fold Change)
A
(A) Hierarchical clustering heatmap of 16 samples from patients with stage I lung adenocarcinoma with vascular invasion. (B) Volcano plot of differentially expressed microRNAs (miRNAs) between the G1 (recurrence+) and G2 (recurrence–) cohorts. (C) Charts indicate the fold-change expression of 79 miRNAs identified as recurrence-related marker candidates
down-regulated
Up-regulated
C
### Chart
| Category | |
|---|---|log 2 (Fold Change)

## Slide 2
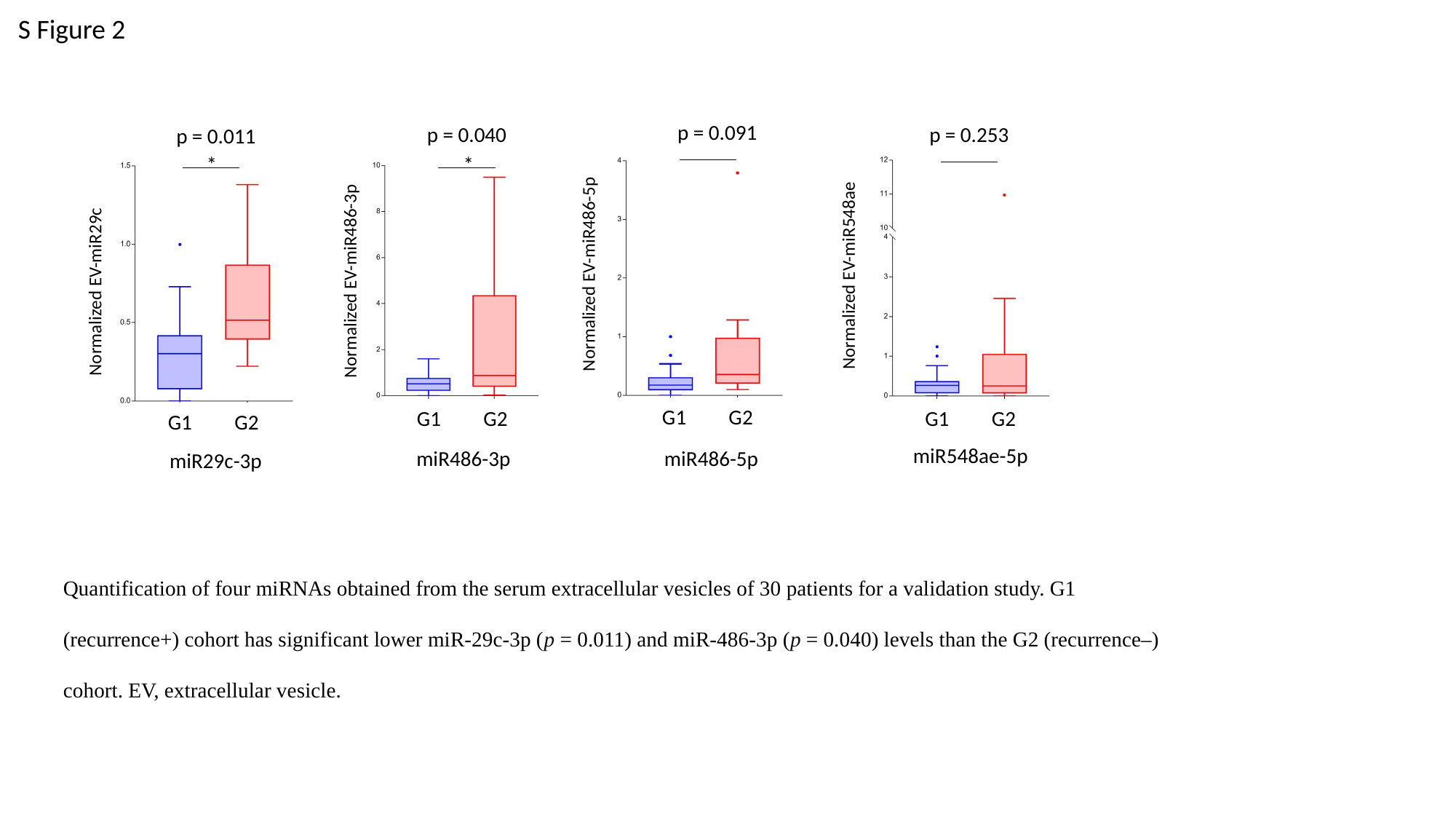

S Figure 2
p = 0.091
p = 0.253
p = 0.040
p = 0.011
*
*
Normalized EV-miR486-5p
Normalized EV-miR548ae
Normalized EV-miR486-3p
Normalized EV-miR29c
G1 G2
G1 G2
G1 G2
G1 G2
miR548ae-5p
miR486-3p
miR486-5p
miR29c-3p
Quantification of four miRNAs obtained from the serum extracellular vesicles of 30 patients for a validation study. G1 (recurrence+) cohort has significant lower miR-29c-3p (p = 0.011) and miR-486-3p (p = 0.040) levels than the G2 (recurrence–) cohort. EV, extracellular vesicle.

## Slide 3
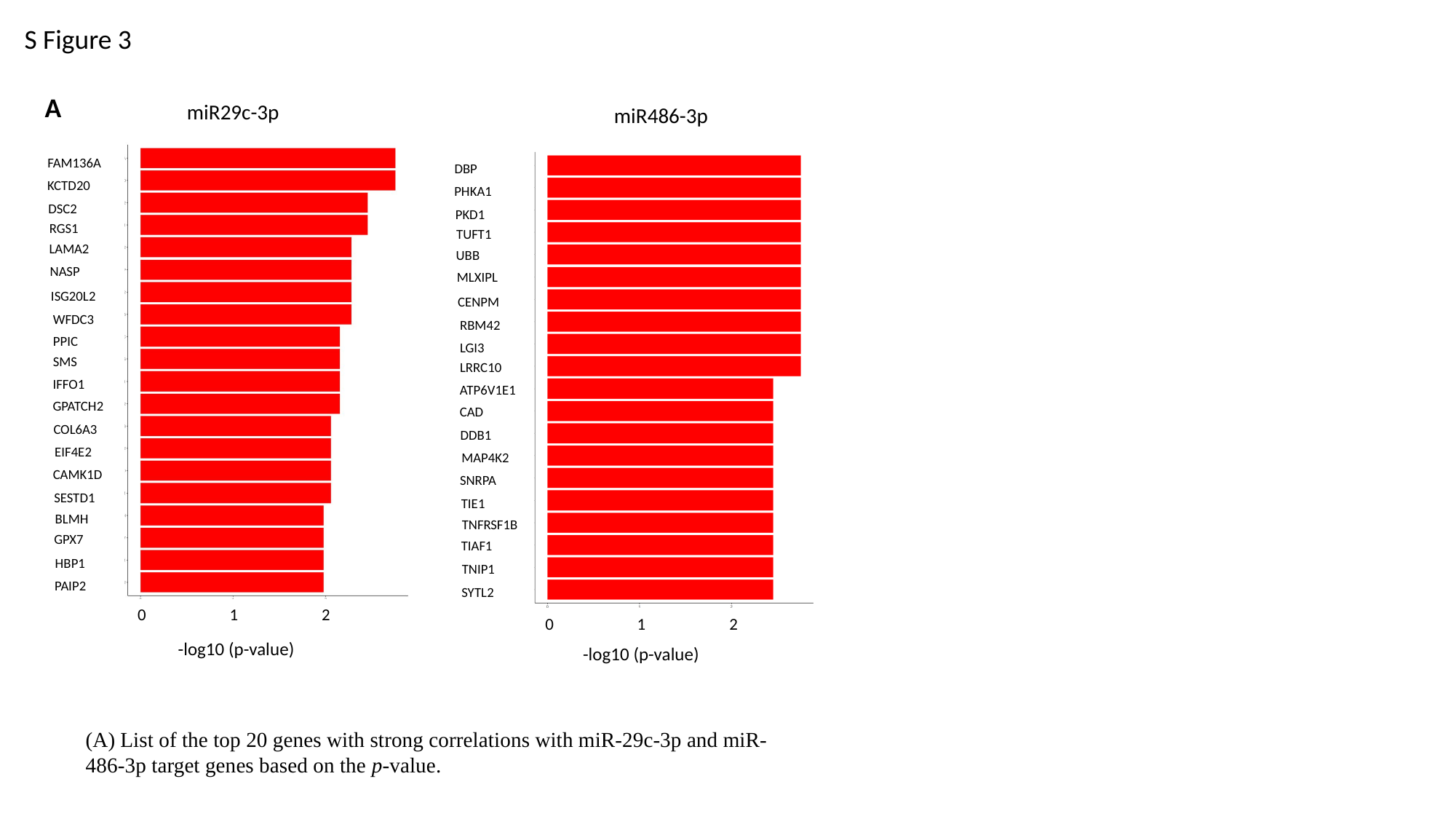

S Figure 3
A
miR29c-3p
miR486-3p
FAM136A
DBP
KCTD20
PHKA1
DSC2
PKD1
RGS1
TUFT1
LAMA2
UBB
NASP
MLXIPL
ISG20L2
CENPM
WFDC3
RBM42
PPIC
LGI3
SMS
LRRC10
IFFO1
ATP6V1E1
GPATCH2
CAD
COL6A3
DDB1
EIF4E2
MAP4K2
CAMK1D
SNRPA
SESTD1
TIE1
BLMH
TNFRSF1B
GPX7
TIAF1
HBP1
TNIP1
PAIP2
SYTL2
0 1 2
0 1 2
-log10 (p-value)
-log10 (p-value)
(A) List of the top 20 genes with strong correlations with miR-29c-3p and miR-486-3p target genes based on the p-value.

## Slide 4
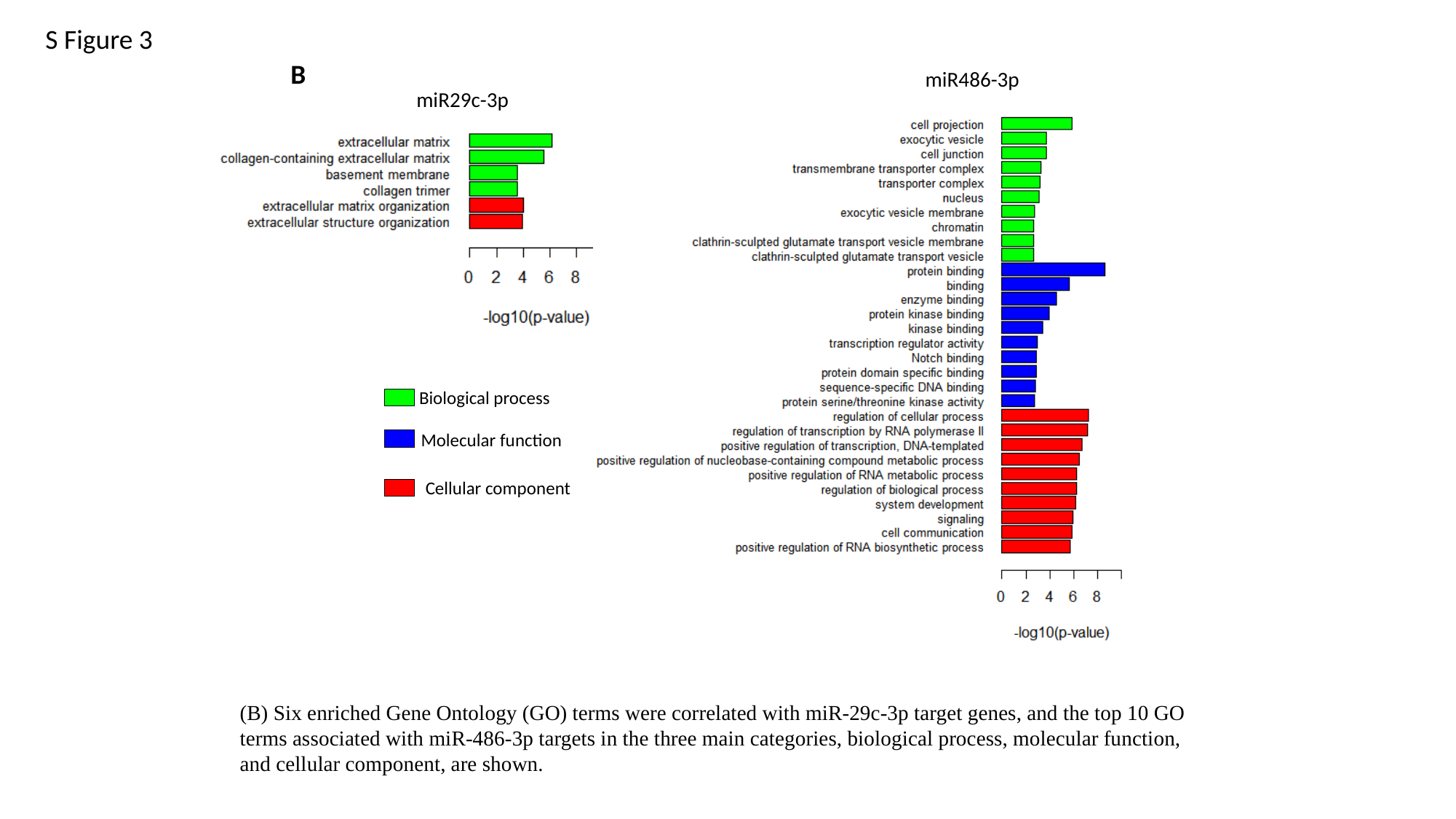

S Figure 3
B
miR486-3p
miR29c-3p
Biological process
Molecular function
Cellular component
(B) Six enriched Gene Ontology (GO) terms were correlated with miR-29c-3p target genes, and the top 10 GO terms associated with miR-486-3p targets in the three main categories, biological process, molecular function, and cellular component, are shown.

## Slide 5
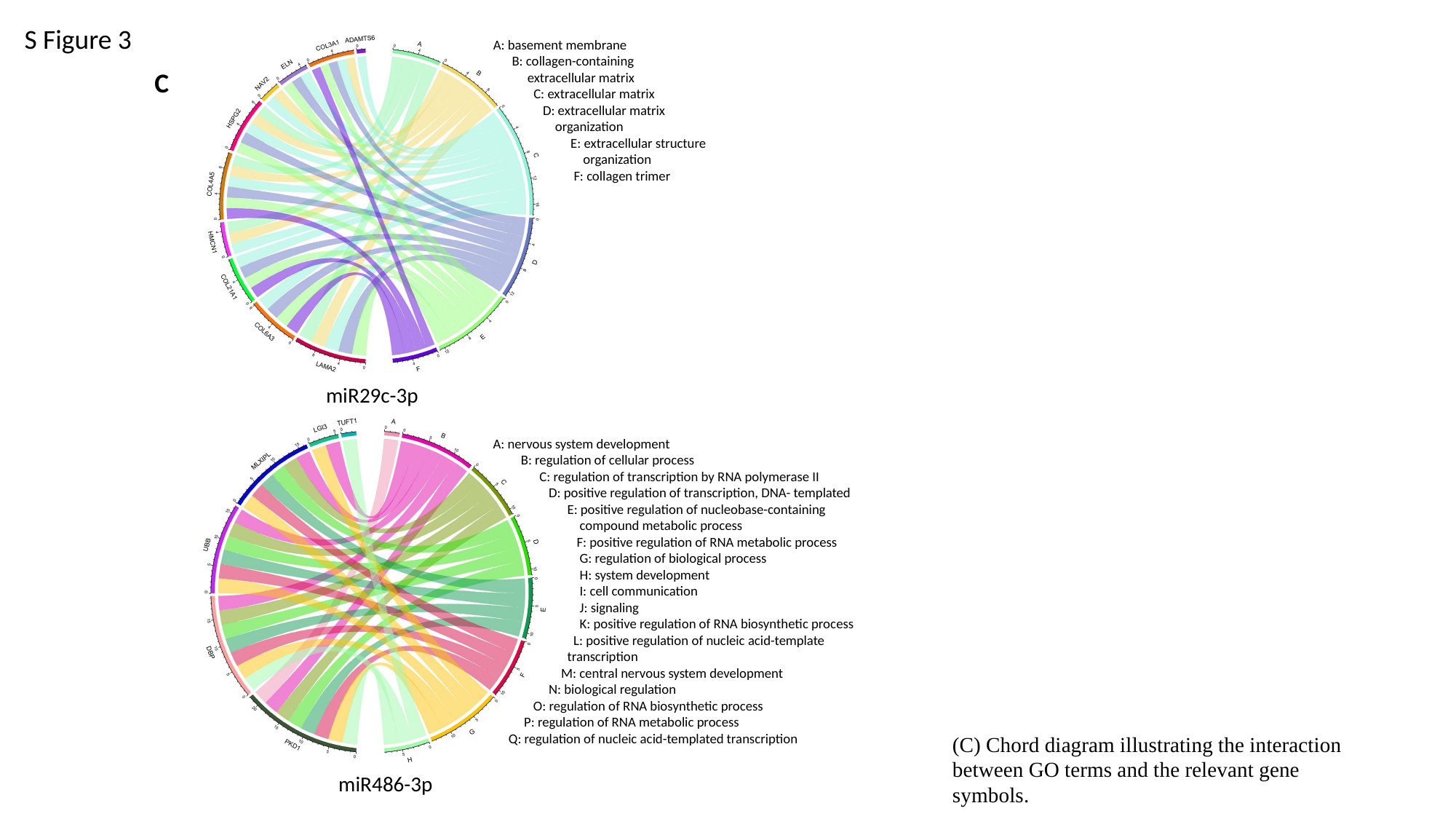

S Figure 3
A: basement membrane
 B: collagen-containing
 extracellular matrix
 C: extracellular matrix
 D: extracellular matrix
 organization
 E: extracellular structure
 organization
 F: collagen trimer
C
miR29c-3p
A: nervous system development
 B: regulation of cellular process
 C: regulation of transcription by RNA polymerase II
 D: positive regulation of transcription, DNA- templated
 E: positive regulation of nucleobase-containing
 compound metabolic process
 F: positive regulation of RNA metabolic process
 G: regulation of biological process
 H: system development
 I: cell communication
 J: signaling
 K: positive regulation of RNA biosynthetic process
 L: positive regulation of nucleic acid-template
 transcription
 M: central nervous system development
 N: biological regulation
 O: regulation of RNA biosynthetic process
 P: regulation of RNA metabolic process
 Q: regulation of nucleic acid-templated transcription
(C) Chord diagram illustrating the interaction between GO terms and the relevant gene symbols.
miR486-3p

## Slide 6
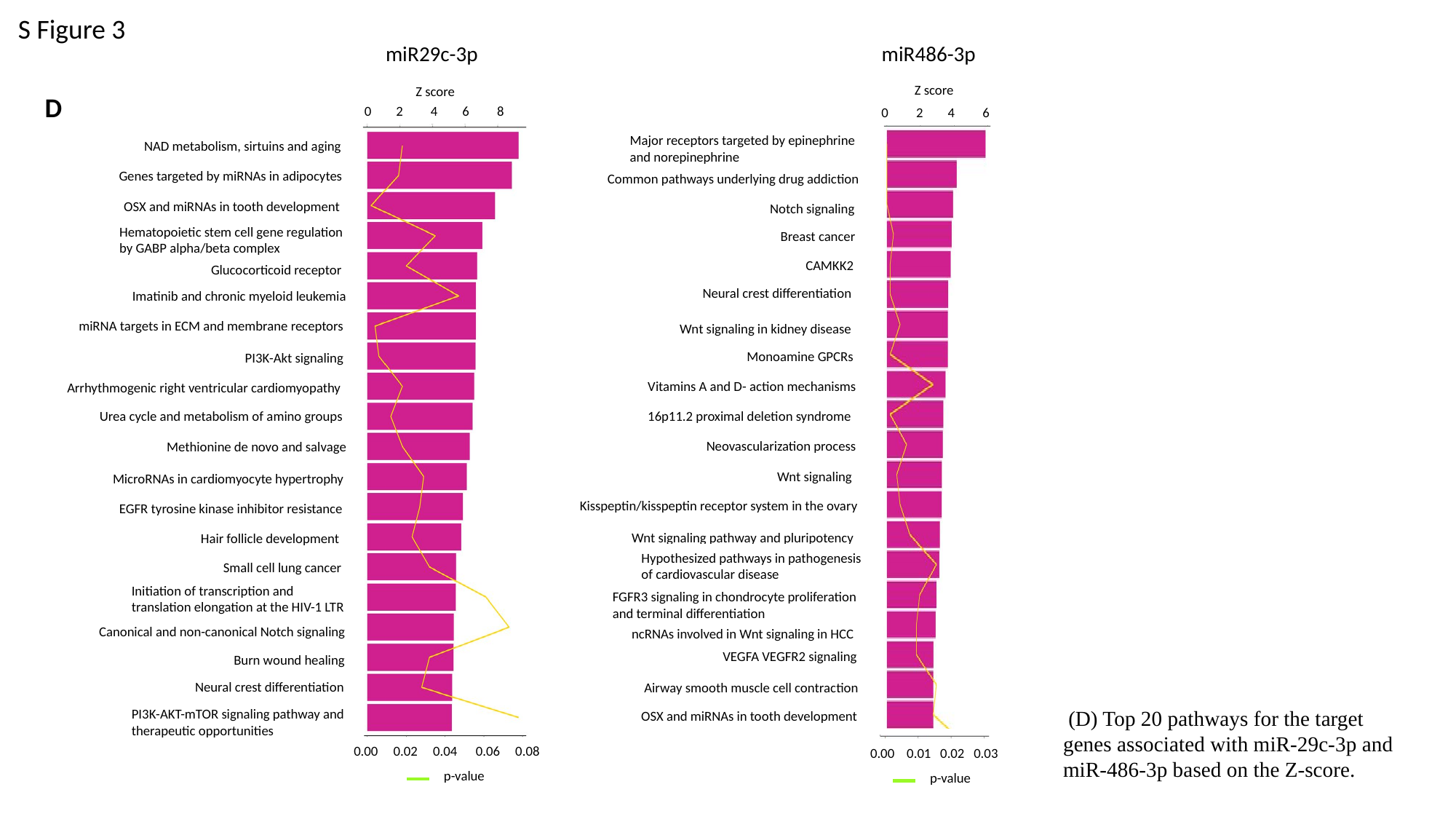

S Figure 3
miR486-3p
miR29c-3p
Z score
Z score
D
0 2 4 6 8
0 2 4 6
Major receptors targeted by epinephrine
and norepinephrine
NAD metabolism, sirtuins and aging
Genes targeted by miRNAs in adipocytes
Common pathways underlying drug addiction
OSX and miRNAs in tooth development
Notch signaling
Hematopoietic stem cell gene regulation
by GABP alpha/beta complex
Breast cancer
CAMKK2
Glucocorticoid receptor
Neural crest differentiation
Interferon type I signaling
Imatinib and chronic myeloid leukemia
miRNA targets in ECM and membrane receptors
Wnt signaling in kidney disease
Monoamine GPCRs
PI3K-Akt signaling
Vitamins A and D- action mechanisms
Arrhythmogenic right ventricular cardiomyopathy
16p11.2 proximal deletion syndrome
Urea cycle and metabolism of amino groups
Neovascularization process
Methionine de novo and salvage
Wnt signaling
MicroRNAs in cardiomyocyte hypertrophy
Kisspeptin/kisspeptin receptor system in the ovary
EGFR tyrosine kinase inhibitor resistance
Wnt signaling pathway and pluripotency
Hair follicle development
Hypothesized pathways in pathogenesis of cardiovascular disease
Small cell lung cancer
Initiation of transcription and translation elongation at the HIV-1 LTR
FGFR3 signaling in chondrocyte proliferation and terminal differentiation
Canonical and non-canonical Notch signaling
ncRNAs involved in Wnt signaling in HCC
VEGFA VEGFR2 signaling
Burn wound healing
Neural crest differentiation
Airway smooth muscle cell contraction
 (D) Top 20 pathways for the target genes associated with miR-29c-3p and miR-486-3p based on the Z-score.
PI3K-AKT-mTOR signaling pathway and therapeutic opportunities
OSX and miRNAs in tooth development
0.00 0.02 0.04 0.06 0.08
0.00 0.01 0.02 0.03
p-value
p-value
