# Supplementary material for: Extracellular vesicle-associated microRNA signatures related to lymphovascular invasion in early-stage lung adenocarcinoma
Source: Sci Rep. 2023 Mar 24;13:4823. doi: 10.1038/s41598-023-32041-5 (PMC10038982; doi:10.1038/s41598-023-32041-5)
Supplement: Supplementary file 2 — Supplementary Tables. [file 41598_2023_32041_MOESM2_ESM.docx]

Supplement Table 1 Characteristics of patients with stage I non-small cell lung cancer from a database (N = 2044)

| Characteristics | Number of patients (%) |
| --- | --- |
| Age, years, median (range) | 69 (20 - 93) |
| Sex, male/ female | 1046 (51)/ 998 (49) |
| Smoking history, yes/ no | 1107 (54)/ 937 (46) |
| SUVmax of tumor, median (range) | 1.9 (0 – 54.9) |
| Clinical stage, IA/ IB/ II/ III | 1549 (76)/401 (20)/ 78 (4)/ 16 (1) |
| Surgical procedure, sublobar resection/ lobectomy | 672 (33)/ 1372 (67) |
| Histology, adenocarcinoma/ non-adenocarcinoma | 1712 (84)/ 332 (16) |
| Pathological tumor size, cm, median (range) | 2.0 (0.4 – 12.0) |
| Pathological stage, IA/ IB | 1483 (73)/ 561 (28) |
| Lymphovascular invasion, positive/ negative | 559 (27)/ 1485 (73) |

SUVmax = the maximum standardized uptake value.

Supplement Table 2 Univariate and multivariate analyses of overall survival in patients with pathological stage I non-small cell lung cancer (N = 2044)

| Variable | Univariate analysis  Hazard ratio (95% CI) | *p*-value |
| --- | --- | --- |
| Age | 1.030 (1.005 – 1.055) | 0.020 |
| Sex (Male vs. Female) | 1.582 (1.018 – 2.463) | 0.041 |
| Smoking history (Yes vs. No) | 1.779 (1.131 – 2,799) | 0.013 |
| SUVmax of tumor | 1.094 (1.069 – 1.120) | < 0.001 |
| Surgical procedure (Lobectomy vs. sublobar) | 1.208 (0.746 – 1.957) | 0.442 |
| Histology (Non-Ad vs. Ad) | 1.923 (1.163 – 3.185) | 0.011 |
| Pathological tumor size | 1.187 (0.990 – 1.424) | 0.064 |
| Pathological stage (IB vs. IA) | 1.934 (1.350 – 2.323) | < 0.001 |
| Lymphovascular invasion (Yes vs. No) | 6.308 (3.976 – 10.006) | < 0.001 |
| Variables | Multivariate analysis  Hazard ratio (95% CI) | *p*-value |
| SUVmax of tumor | 1.043 (1.008 – 1.079) | 0.017 |
| Pathological tumor size | 1.357 (1.060 – 1.733) | 0.015 |
| Pathological stage (IB vs. IA) | 2.427 (1.428 – 4.124) | 0.001 |
| Lymphovascular invasion (Yes vs. No) | 4.134 (2.431 – 7.031) | < 0.001 |

CI = confidence interval, SUVmax = the maximum standardized uptake value., Ad = adenocarcinoma.

Supplement Table 3 Univariate and multivariate analyses of recurrence-free survival in patients with pathological stage I non-small cell lung cancer (N = 2044)

| Variable | Univariate analysis  Hazard ratio (95% CI) | *p*-value |
| --- | --- | --- |
| Age | 1.058 (1.043 – 1.074) | < 0.001 |
| Sex (Male vs. Female) | 2.611 (2.004 – 3.401) | < 0.001 |
| Smoking history (Yes vs. No) | 2.989 (2.258 – 3.957) | < 0.001 |
| SUVmax of tumor | 1.088 (1.066 – 1.093) | < 0.001 |
| Surgical procedure (Lobectomy vs. sublobar) | 1.087 (0.845 – 1.399) | 0.517 |
| Pathological stage (IB vs. IA) | 3.130 (2.415 – 3.975) | < 0.001 |
| Pathological tumor size | 1.289 (1.179 – 1.407) | < 0.001 |
| Histology (Non-Ad vs. Ad) | 3.175 (2.469 – 4.082) | 0.011 |
| Lymphovascular invasion (Yes vs. No) | 4.376 (3.475 – 5.576) | < 0.001 |
| Variables | Multivariate analysis  Hazard ratio (95% CI) | *p*-value |
| Age | 1.048 (1.032 – 1.063) | < 0.001 |
| Smoking history (Yes vs. No) | 1.657 (1.140 – 2.408) | 0.008 |
| SUVmax of tumor | 1.024 (1.004 – 1.044) | 0.020 |
| Surgical procedure (Lobectomy vs. sublobar) | 1.563 (1.195 – 2.045) | 0.001 |
| Pathological stage (IB vs. IA) | 1.771 (1.350 – 2.323) | < 0.001 |
| Lymphovascular invasion (Yes vs. No) | 2.664 (2.000 – 3.548) | < 0.001 |

CI = confidence interval, SUVmax = the maximum standardized uptake value., Ad = adenocarcinoma.
